# Persistent Post-Burn Pruritus Following Antihistamine Initiation: A Retrospective Longitudinal Study at a Single Italian Pediatric Burn Center

**DOI:** 10.3390/medsci14060634

**Published:** 2026-10-02

**Authors:** Biagio Nicolosi, Emanuele Buccione, Felice Curcio, Lorenzo Drago, Salvatore Angileri, Pedro Miguel Garcez Sardo, Alexandre Marques Rodrigues, Jenifer Adriana Domingues Guedes, Gianluca Azzellino, Greta Ghizzardi, Guido Ciprandi, Rosario Caruso

**Affiliations:** 1 Department of Health Professions, Meyer Children’s Hospital IRCCS, 50139 Florence, Italy; 2 AISLeC—Italian Nursing Association for the Study of Skin Lesions, 47923 Rimini, Italy; 3 Pediatric Intensive Care Unit, Local Health Authority of Pescara, 65124 Pescara, Italy; 4 Faculty of Medicine and Surgery, University of Sassari, 07100 Sassari, Italy; felice.curcio@aouss.it; 5 UOC Clinical Pathology Laboratory with Specialized Areas, IRCCS MultiMedica, 20138 Milan, Italy; lorenzo.drago@unimi.it; 6 Department of Biomedical Sciences for Health, University of Milan, 20133 Milan, Italy; rosario.caruso@unimi.it; 7 Clinical and Cellular Pathophysiology Research Unit, Meyer Children’s Hospital IRCCS, 50139 Florence, Italy; salvatore.angileri@meyer.it; 8 School of Health Sciences, University of Aveiro, 3810-193 Aveiro, Portugal; pedro.sardo@ua.pt (P.M.G.S.); alexandre.rodrigues@ua.pt (A.M.R.); 9 Institute of Biomedicine (iBiMED), University of Aveiro, 3810-193 Aveiro, Portugal; 10 Center for Health Studies and Research, University of Coimbra (CEISUC), 3000-548 Coimbra, Portugal; 11 Pediatric Emergency Department, Local Health Unit of the Aveiro Region, 3880-113 Ovar, Portugal; jenifer.guedes@ua.pt; 12 Department of Life, Health and Environmental Sciences, University of L’Aquila, 67100 L’Aquila, Italy; gianluca.azzellino@graduate.univaq.it; 13 Adriatic District Area, Local Health Authority of Teramo, 64100 Teramo, Italy; 14 Directorate of Nursing and Allied Health Professions, ASST Lodi, 26900 Lodi, Italy; greta.ghizzardi@asst-lodi.it; 15 Complex Unit of Plastic Surgery, University of Padua, 35122 Padua, Italy; guido.ciprandi@unipd.it; 16 Health Professions Research and Evidence Transfer Unit, IRCCS MultiMedica, 20099 Sesto San Giovanni, Italy

**Keywords:** burns, pruritus, child, antihistamines, nursing assessment

## Abstract

**Background/Objectives:** An operationally defined unchanged itch-score sequence may indicate persistent symptoms rather than symptom resolution. We described pruritus following antihistamine initiation in children with burns and examined how its apparent stabilization depended on the analytical definition. **Methods:** This retrospective single-center study included 247 children. Daily scores were reconstructed from clinical charts using IMS-derived and TPIS-derived coding frameworks. Analyses added during revision examined alternative stability rules, candidate breakpoints, separate tool-specific trajectories and missingness. **Results:** Mean normalized itch decreased from 0.836 on Day 0 to 0.628 on Day 2 and 0.605 on Day 3 and then ranged from 0.591 to 0.608 through Day 14. All 3210 observed scores remained above zero, including all 197 available Day-14 assessments; 50 Day-14 scores were missing. A post hoc rule requiring three unchanged consecutive daily scores after at least one category of improvement was met by 177/247 children (71.7%), with a conditional median confirmation day of 4 (IQR 3–5). Alternative rules gave medians of 3 and 5. Candidate model comparisons favored Day 2; age/tool trajectories differed (*p* < 0.001). Adjusted models included 2654 observations from 205 children. **Conclusions:** The recorded trajectory showed early reduction followed by little change in mean itch, with persistent symptoms in observed assessments. The timing of operational score-stability events depended on the definition applied. Neither a drug effect nor a validated clinical reassessment threshold can be inferred.

## 1. Introduction

Post-burn pruritus is a frequent and persistent complication in children. In a multicenter cohort, 93% of pediatric burn survivors had pruritus at hospital discharge, and 63% still reported symptoms two years after injury [1]. These observations show why a decrease in itch intensity should be distinguished from symptom resolution.

Itch may interfere with sleep, daily activities and recovery. Pediatric itch interference measures have been developed using both self-report and proxy-report approaches, reflecting the need to consider functional burden as well as symptom intensity [2]. Repeated clinical assessment is particularly important when itch remains present despite an apparently stable score.

Post-burn itch involves interacting inflammatory and neural pathways [3]. A change in symptom severity during healing does not by itself identify the mechanism responsible for that change. The timing of treatment, wound recovery and concurrent care must therefore be considered when interpreting a clinical trajectory.

Antihistamines are used in post-burn itch management, but persistent symptoms have prompted investigation of other approaches [3]. A systematic review of neuropathic agents found evidence suggesting a role for gabapentinoids while also identifying limitations in the available studies [4].

The 2024 Cochrane review found that evidence for post-burn pruritus interventions was generally of moderate-to-low certainty, with small studies and risks of bias [5]. Evidence on treatment effects should not be conflated with evidence defining a particular time for reassessment in children.

The course of itch following antihistamine initiation may help describe symptom burden during recovery. However, improvement observed after treatment starts cannot by itself establish a treatment effect. Wound healing, concurrent care, regression to the mean and treatment may all contribute to the observed trajectory. A useful reassessment schedule therefore requires evidence beyond a temporal association.

This study aimed to describe the daily course and persistence of reconstructed pruritus scores during the first 14 days following antihistamine initiation and to examine how operational definitions and model specification affected the apparent timing of stabilization.

## 2. Materials and Methods

### 2.1. Study Design

This retrospective, single-center observational study examined the course of post-burn pruritus following antihistamine initiation. Clinical management was not assigned by the study, and there was no untreated comparison group. The analyses are descriptive and exploratory; they do not estimate antihistamine efficacy. Reporting follows the Strengthening the Reporting of Observational Studies in Epidemiology (STROBE) guidelines [6].

### 2.2. Setting and Participants

The study was conducted at Meyer Children’s Hospital IRCCS, a tertiary pediatric burn center in Florence, Italy. Data were obtained from the medical and nursing records of children admitted between January 2023 and December 2024. All 247 available records for the study population were included, without sampling a subset. The clinical observations originated from patient charts.

Patients were eligible if they were between 6 months and 18 years of age, had burns involving more than 10% of the total body surface area (TBSA) with a depth of at least superficial partial thickness, developed post-burn pruritus during re-epithelialization or the chronic healing phase, received systemic antihistamine therapy for at least 72 h, and had at least one baseline and two follow-up pruritus assessments available for longitudinal evaluation.

The eligibility criteria excluded pruritus attributable to other causes, concomitant systemic antipruritic treatment with gabapentinoids, tricyclic antidepressants or systemic corticosteroids, documented contraindications to antihistamines, and records without sufficient baseline information to establish eligibility. Inclusion of a child did not require complete daily follow-up or complete model covariates. Missing daily assessments were retained as missing; exclusions from adjusted models were handled separately from cohort inclusion.

### 2.3. Pruritus Assessment

Pruritus was not routinely assessed with standardized instruments at the study center. Scores were therefore reconstructed retrospectively from medical and nursing documentation. These reconstructed categories should not be interpreted as prospectively administered, validated scale measurements.

The Itch Man Scale (IMS) and the Toronto Pediatric Itch Scale (TPIS) provided the reference framework for children aged ≥6 years and younger children, respectively. Both instruments have published validation evidence from English-language clinical settings [7,8]. These studies support their original uses, rather than the validity of retrospectively assigning categories from narrative records. Narrative descriptions were mapped to categories reflecting the domains of each instrument.

The English-language instruments were translated into Italian and underwent linguistic adaptation with a final review by five experts. This was a language-adaptation process, not a psychometric validation of the Italian versions. Methodological guidance distinguishes translation and expert review from pretesting and subsequent evaluation of measurement properties [9]. The present study did not establish cross-cultural validity, reliability or responsiveness of the Italian versions. The retrospective coding framework introduces a further measurement limitation and is described separately in Supplementary File S1.

The coding algorithm was based on the conceptual domains of the original IMS and TPIS and linked predefined clinical descriptors—including patient-reported itching, scratching behavior, sleep disturbance, interference with daily activities, nursing observations, and the need for therapeutic interventions—to the corresponding score category. To minimize misclassification bias, only clinical records meeting predefined criteria for completeness and internal consistency were coded; incomplete, contradictory, or non-interpretable documentation was classified as missing rather than being assigned a score.

Retrospective score attribution was performed by an experienced wound-care nurse. A second investigator reviewed coding ambiguities, which were resolved by consensus. This review served as a quality-control step to support consistent application of the coding framework; it was not a formal independent reliability study. Separate pre-consensus ratings were not collected. Consequently, weighted kappa and its confidence interval could not be calculated, and the reproducibility of score reconstruction remains unquantified. No new independent recoding was possible during revision.

IMS-derived and TPIS-derived scores were described and modelled separately on their original ranges (0–4 and 0–3, respectively). For secondary pooled analyses, scores were divided by their respective maxima. This rescaling puts values on a common numerical range; it does not establish measurement equivalence. Tool assignment was determined by age, so differences between tools cannot be separated from differences between age groups. Daily scores followed the original worst-of-day extraction rule.

### 2.4. Data Collection

The standardized electronic case report form in REDCap^®^ contained demographic characteristics; burn etiology, extent, depth, anatomical sites and time to re-epithelialization; antihistamine exposure and modifications; and recorded care variables. Burn extent was expressed as percentage total body surface area (TBSA). Depth was coded as superficial partial thickness, deep partial thickness or full thickness, corresponding to the anatomical classification described by Jeschke et al. [10]. The recorded TBSA and depth were used directly; burn size and depth were not reassessed during this analysis.

Standard pediatric burn assessment uses age-adjusted surface-area mapping, such as the Lund–Browder chart, together with anatomical depth and site documentation. Healing assessment distinguishes open wounds, re-epithelialization and scar maturation; graft status and timing are separate clinical features [10]. In this dataset, TBSA and depth entered the adjusted models. Anatomical sites were recorded as nonexclusive indicators, a summarized in Table 1, but were not included as model covariates. Time to re-epithelialization was recorded, but daily healing phase was not modelled; days after antihistamine initiation cannot substitute for days after injury or healing stage. Skin-graft status, graft dates and donor-site itch were not available as analysis variables.

Wound care is ordinarily characterized by cleansing or debridement, dressing material, change frequency and procedural discomfort. Infection assessment distinguishes clinical wound findings from colonization, with microbiological investigation performed when indicated [10]. The models included dressing-change and painful-procedure counts, physiotherapy-session counts, and a binary indicator of local infection or complications. These were follow-up summaries, not daily exposures; the composite complication field did not distinguish infection from other local problems. Dressing products, debridement methods, infection onset and microbiological confirmation were not modelled.

Skin dryness and barrier care require separate assessment from itch severity; moisturizers and other comfort measures may accompany pharmacological treatment [10]. The dataset did not contain structured exposure measures for xerosis, skin hydration, comfort measures or patient/caregiver education. Analgesic adjustment was limited to recorded opioid use and sedation/drowsiness, without daily doses, administration times or a complete non-opioid analgesic history. Consequently, neither these care effects nor the antihistamine contribution could be isolated. Supplementary File S3 specifies the limits of covariate control.

### 2.5. Outcomes

The descriptive outcome was the daily course of reconstructed itch scores. Individual operational score-stability events were examined as exploratory outcomes. The original plateau definition was not treated as a validated clinical endpoint.

The original definition—less than a one-point reduction for two consecutive days—did not specify how to handle worsening or missing observations and has no validated minimal clinically important difference. We therefore applied explicit post hoc rules: (A) three identical scores on three consecutive calendar days; (B) rule A with a reduction of at least one category from Day 0; and (C) four identical consecutive daily scores after that reduction. The event day was the last day required to confirm the rule, not the start of the unchanged sequence. Missing days interrupted a sequence; nonconsecutive observations were not joined or imputed. Worsening interrupted a sequence, and a later qualifying sequence could still count. Rule A allowed unchanged low scores without prior improvement; rules B and C required improvement from baseline. A sensitivity version of rule B excluded zero scores. Children without a qualifying observed sequence by Day 14 were classified as having no observed stability event, and the absence of later stabilization was not assumed. These are operational score-stability rules identifying the first occurrence of a qualifying score sequence; they are not MCID thresholds and do not demonstrate sustained score stability or clinical stabilization thereafter.

Additional descriptive outcomes included antihistamine exposure, treatment modifications and documented care-related events. Associations following a treatment switch were considered exploratory because switching was not randomized and its indications could be related to symptom severity.

### 2.6. Bias

Potential biases included selection through the availability of repeated assessments, retrospective score reconstruction, confounding by indication and informative assessment frequency. Natural recovery and concurrent care cannot be separated from a drug effect. Adjustment used recorded patient characteristics and care variables, but the latter were patient-level follow-up summaries rather than daily time-varying measurements. Such adjustment does not remove residual confounding and may condition on consequences of the clinical course.

### 2.7. Study Size

The sample size was determined by the fixed number of available records for the study population during the January 2023–December 2024 period. All 247 were included; no subset was selected, and no target sample size was used. This was a census of the available study records, not a probability sample of pediatric burn patients. STROBE guidance recommends explaining a fixed available sample when it determines study size and reporting the precision achieved rather than retrospective power calculations [11]. Accordingly, no a priori power calculation was performed; estimates are accompanied by 95% confidence intervals. Repeated daily records are clustered within children and do not constitute independent participants. Adjusted analyses included 205 children with complete covariates; the other 42 remained in the descriptive cohort.

### 2.8. Statistical Analysis

Continuous variables are summarized as mean ± standard deviation (SD) or median and interquartile range (IQR), according to data distribution. Categorical variables are presented as frequencies and percentages.

Analyses covered Day 0 through Day 14 following antihistamine initiation, including observations after treatment ended when present. This time axis describes follow-up from initiation and does not imply continuous treatment throughout the 14 days. Stability events were recalculated directly from the wide-format daily scores; the stored plateau estimate was not used to define the revised events or population breakpoints.

Daily means, standard deviations and pointwise 95% confidence intervals were calculated from available observations. IMS and TPIS were examined separately on their original scales. Pooled normalized scores were used only as a secondary numerical summary, with no assumption that equal normalized values represent equal clinical severity.

For comparison with the original specification, a segmented linear mixed-effects model with a patient-specific random intercept was fitted by maximum likelihood at a fixed Day-7 breakpoint. Fixed effects comprised day, an indicator for day ≥7, and max (0, Day −7), together with age, sex, weight, TBSA, burn depth, burn etiology, opioid use, sedation, dressing-change count, painful-procedure count, physiotherapy-session count and local complications. Reference categories were female sex, deep partial-thickness burns and chemical burns. The hinge coefficient denotes a change in slope; the post-break slope is the sum of the day and hinge coefficients, with its standard error calculated from their covariance. The level-change coefficient permits a discontinuity and is not evidence of an abrupt biological event. Separate fits on the original IMS and TPIS scales used the same specification. A pooled Gaussian GEE with exchangeable working correlation and robust standard errors tested the three tool-by-time terms jointly. These terms describe age/tool differences rather than isolated instrument effects.

Exploratory breakpoint comparisons fitted discontinuous models at Days 2–12 on the same observations and covariates; a Day-1 discontinuous model was excluded because its pre-break segment contained only one time point. Continuous segmented models, without a level-change term, were also compared at Days 1–12. Smaller Akaike information criterion (AIC) values indicated better fit within each model family. This candidate-grid comparison does not provide a validated clinical threshold or a confidence interval for breakpoint timing. Missing outcomes and covariates were not imputed for regression; adjusted analyses were restricted to observed outcomes with complete model covariates. Likelihood-based inference assumes outcome missingness is an ignorable conditional on the model, an assumption that cannot be verified from observed data alone. A logistic GEE with patient-level robust standard errors examined missingness on Days 1–14 when the preceding day was observed, using day, preceding normalized itch, age, sex, TBSA and burn depth. As an extreme descriptive sensitivity analysis, daily means were bounded by assigning every missing score first the scale minimum and then the maximum. These bounds are not imputations or confidence intervals.

All statistical analyses, including the reanalyses performed during revision, were conducted using IBM SPSS Statistics, version 31.0 (IBM Corp., Armonk, NY, USA). Two-sided *p*-values and 95% confidence intervals are reported. Analyses introduced during revision, including alternative stability definitions, breakpoint comparisons, interactions and missingness modelling, were exploratory and were not prespecified. No adjustment for multiple testing was applied.

Population-level segmentation and individual operational score-stability events answer different questions. A population breakpoint indicates a change in the fitted mean trajectory, whereas an individual score-stability confirmation day indicates the first day on which a specified score sequence is confirmed. Neither quantity identifies the end of a drug effect.

### 2.9. Institutional Review Board Statement

The study was conducted in accordance with the Declaration of Helsinki and applicable national and European data protection legislation. The study protocol was approved by the Tuscany Regional Pediatric Ethics Committee (Comitato Etico Regione Toscana–Pediatrico; protocol code 126/2025; approval date: 26 June 2025).

## 3. Results

### 3.1. Study Population

The dataset contained 247 children, with a mean age of 9.6 ± 4.9 years; 52.6% were male. Mean TBSA was 27.1 ± 10.9%. Superficial partial-thickness burns were the most frequent depth category, and 81.0% of burns were coded as thermal. The database did not distinguish scald injuries from other thermal mechanisms. Baseline characteristics are summarized in Table 1.

### 3.2. Antihistamine Exposure

Initial antihistamine treatment characteristics are summarized in Table 2. Second-generation antihistamines were prescribed in the majority of patients, with levocetirizine (25.5%), desloratadine (20.2%), cetirizine (18.2%), and loratadine (14.2%) representing the most frequently used agents. First-generation antihistamines, including hydroxyzine (11.7%) and chlorpheniramine (10.1%), were prescribed less frequently.

Mean antihistamine treatment duration was 11.5 ± 1.7 days, with a mean of 18.3 ± 13.9 recorded doses (Table 3). Treatment started a mean of 5.4 ± 2.5 days after injury. A switch or addition of another antihistamine was recorded in 45 children (18.2%). The recorded treatment interval was shorter than the 14-day observation window for many children.

### 3.3. Longitudinal Course of Pruritus

**Figure 1:**
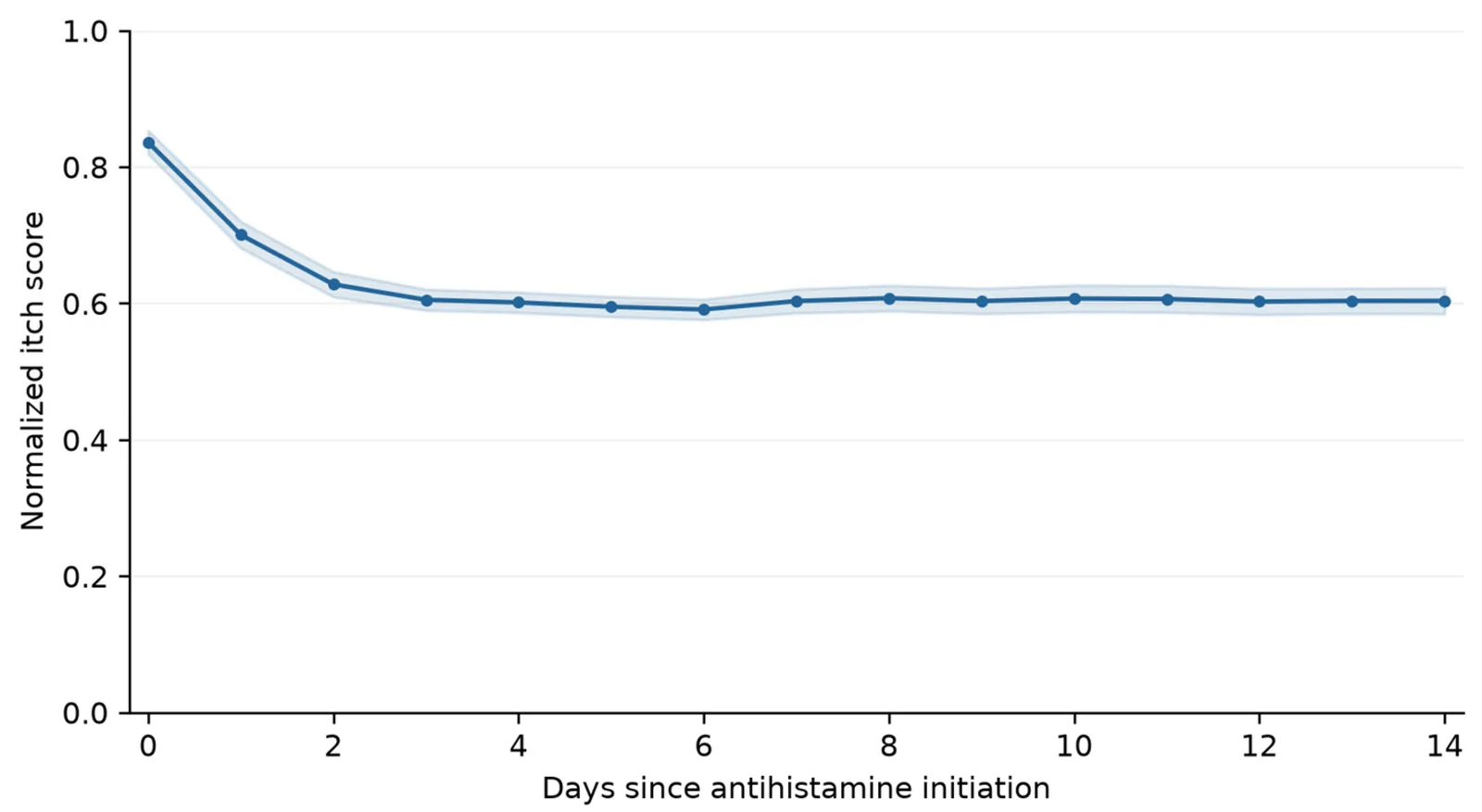
Daily mean normalized itch following antihistamine initiation, with pointwise 95% confidence intervals based on observed scores. Rescaling does not establish equivalence between IMS and TPIS. No clinical reassessment window is assumed.

Daily scores are shown in Table 4 and Figure 1. Mean normalized itch was 0.836 ± 0.139 on Day 0, 0.700 ± 0.153 on Day 1, 0.628 ± 0.141 on Day 2 and 0.605 ± 0.116 on Day 3. Most of the observed mean reduction therefore occurred within the first two days following antihistamine initiation.

From Day 3 through Day 14, daily means ranged from 0.591 to 0.608. All 3210 observed scores were above zero (minimum recorded category, 2). Thus, no symptom-free assessment was recorded during the observation window. On Day 14, pruritus remained recorded in all 197 assessed children (197/197 available assessments; 79.8% of the cohort), while 50/247 assessments were missing. These findings describe persistence in the available records and do not establish the symptom status of children on unobserved days. The mean curve did not show a new onset of stabilization specifically at Days 7–8.

### 3.4. Sensitivity to the Definition of Operational Score Stability

**Figure 2:**
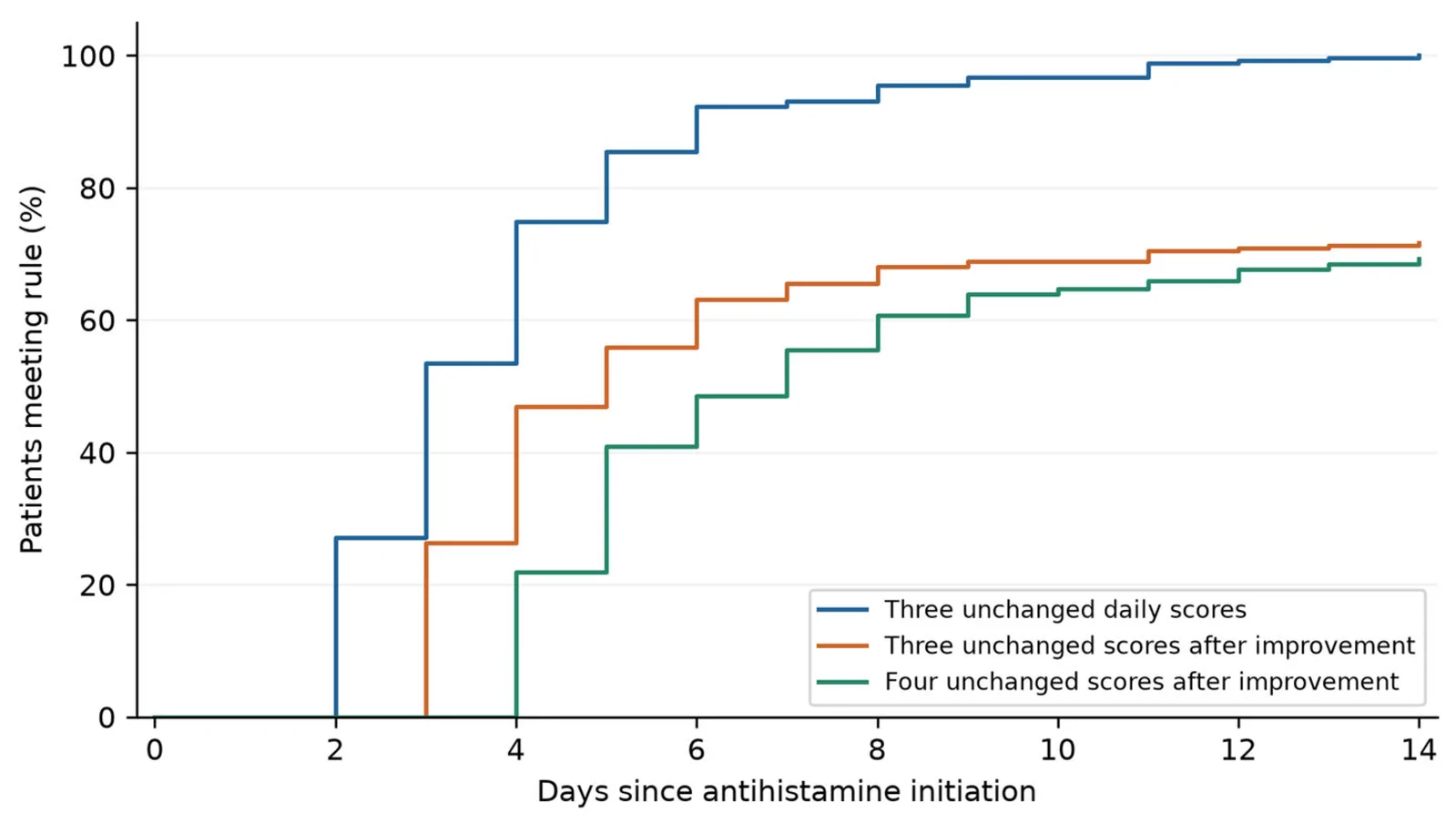
Cumulative proportion of all 247 children meeting each operational score-stability rule by its confirmation day. Missing days interrupt sequences. Curves show the first observed qualifying score-stability events, not Kaplan–Meier estimates, sustained score stability, or clinical stabilization.

The original database field for plateau day did not reproduce the explicit rules applied to the daily scores. For rule A, the confirmation day agreed with the stored plateau day in only 4 of 247 children. The stored distribution was therefore replaced by recalculated operational score-stability events (Table 5; Figure 2).

Three identical scores on consecutive days were observed in all 247 children, with a median confirmation day of 3 (IQR 2–4.5). Requiring at least one category of prior improvement reduced the number meeting the rule to 177/247 (71.7%); the median of Day 4 (IQR 3–5) applies only to these 177 children. This rule was introduced during revision and was not prespecified or clinically validated. Requiring four unchanged daily scores after improvement identified 171/247 children (69.2%), with a conditional median of 5 (IQR 4–7). Excluding zero-score sequences did not change the results because all observed scores were positive.

For the three-score improvement rule, 70 children had no qualifying observed sequence by Day 14. Missing assessments could delay or prevent confirmation, and an event did not require scores to remain stable thereafter. The revised events extended through Day 14; neither the original “no plateau beyond Day 12” statement nor a specific Days 7–8 window was supported by these rules.

### 3.5. Stratified Analyses

**Figure 3:**
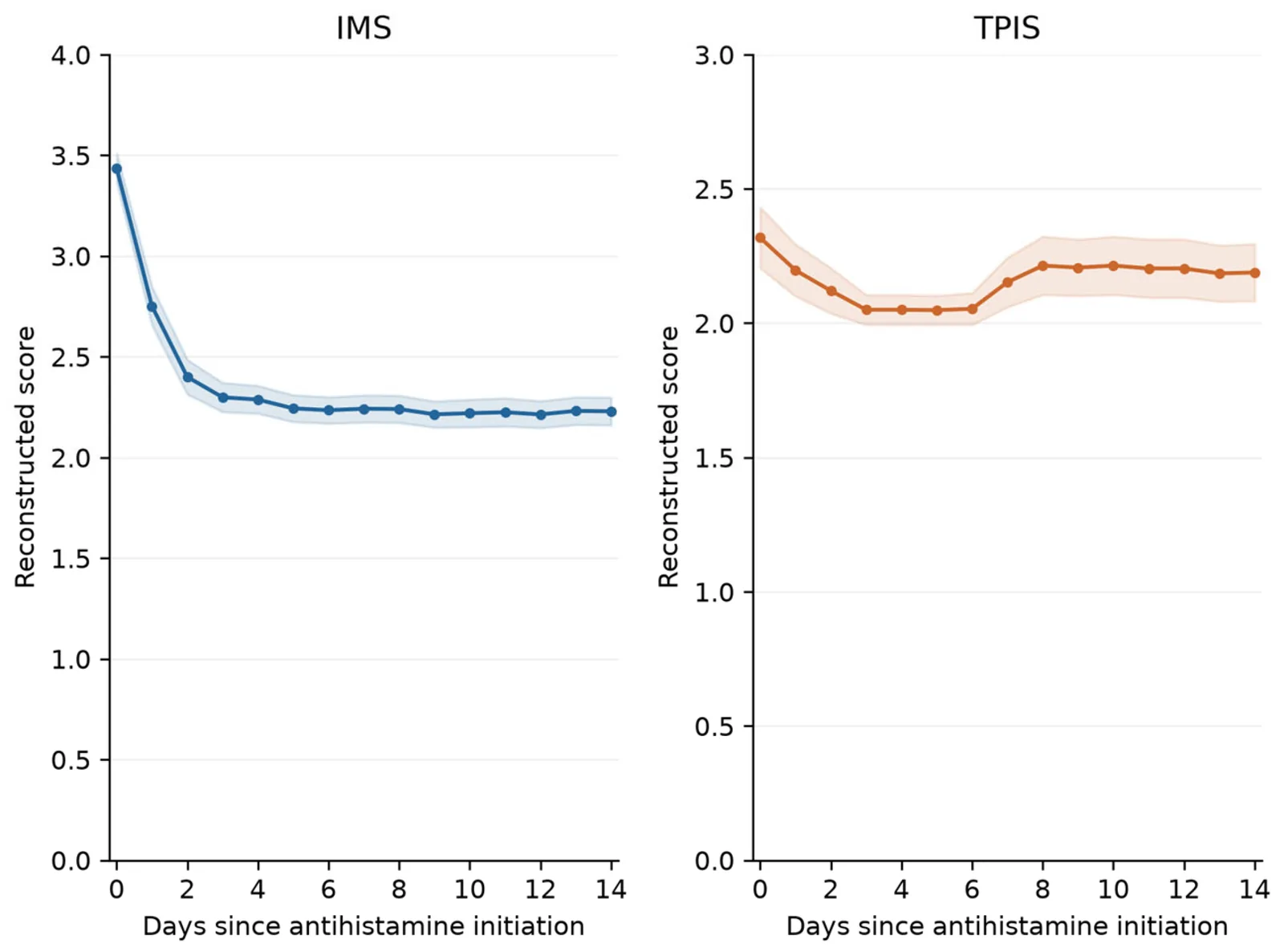
Daily mean reconstructed IMS and TPIS scores on their original scales, with pointwise 95% confidence intervals. Panels have different score ranges; tool assignment is determined by age.

**Figure 4:**
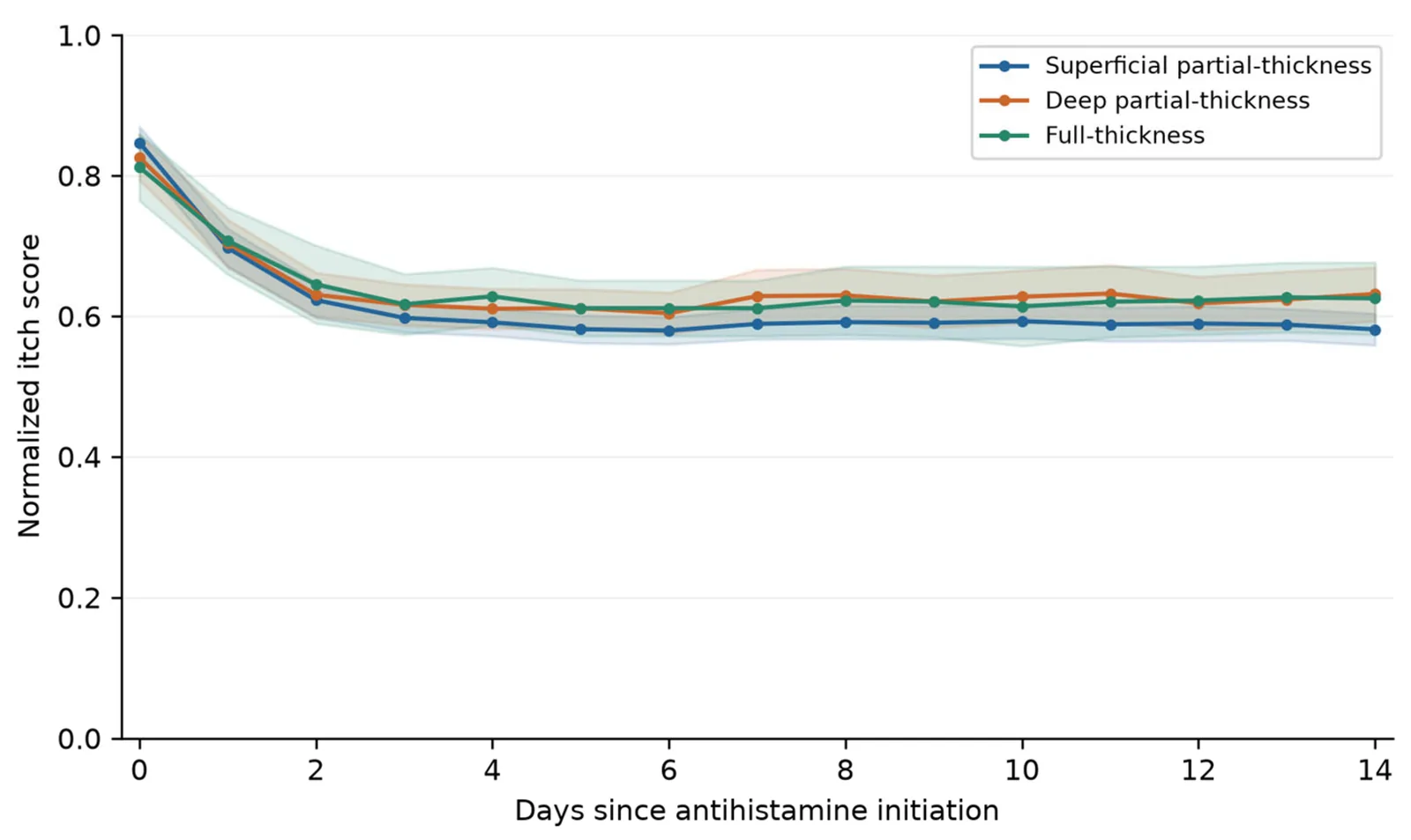
Descriptive normalized itch trajectories by burn depth, with pointwise 95% confidence intervals. Curves do not establish equivalence between depth groups.

**Figure 5:**
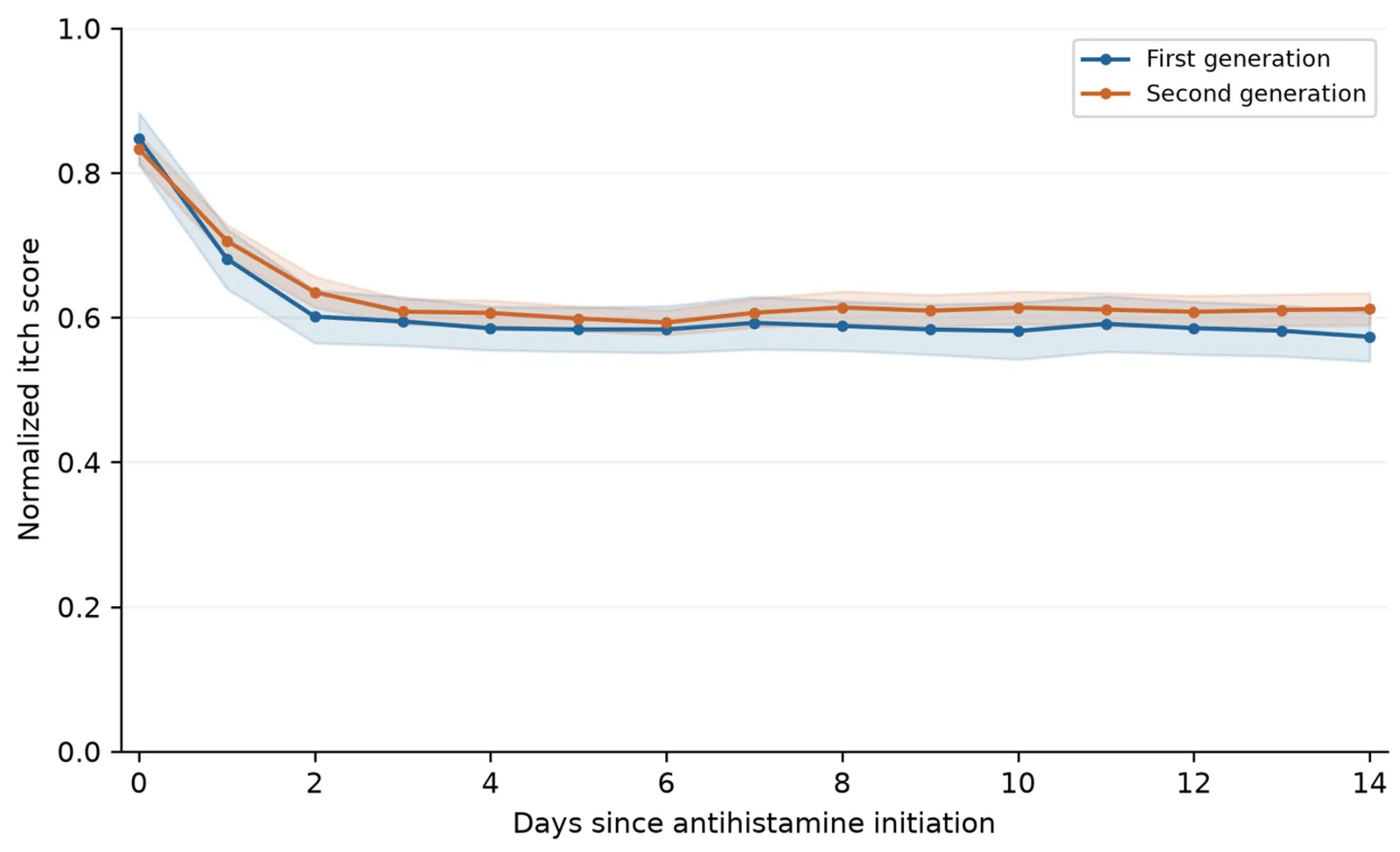
Descriptive normalized itch trajectories by initial antihistamine generation, with pointwise 95% confidence intervals. Treatment allocation was not randomized.

There were 181 children assessed within the IMS framework and 66 within the TPIS framework. Figure 3 presents their reconstructed scores on the original scales; Figure 4 and Figure 5 show descriptive pooled curves by burn depth and antihistamine generation.

The joint age/tool-by-time interaction was significant (Wald χ^2^ = 54.54, 3 df; *p* < 0.001), indicating different trajectories on the normalized scale. Separate adjusted models included 153 IMS children (1968 observations) and 52 TPIS children (686 observations). Pre-Day-7 slopes were −0.1731 IMS categories/day (95% CI −0.1829 to −0.1633) and −0.0429 TPIS categories/day (−0.0565 to −0.0293). Post-Day-7 slopes were 0.0003 (−0.0082 to 0.0088) and 0.0015 (−0.0101 to 0.0132), respectively. These conditional slopes do not establish measurement equivalence or identify the optimal breakpoint. Burn-depth and generation plots are descriptive and do not demonstrate subgroup equivalence.

### 3.6. Segmented Mixed-Effects Model

The fixed-Day-7 mixed-effects model included 2654 observations from 205 children with complete covariates (Table 6). Its pre-break slope was −0.0358/day and the change in slope was +0.0361/day. The resulting post-break slope was +0.0002/day (95% CI −0.0018 to +0.0022; *p* = 0.817). The positive level-change estimate reflects the permitted discontinuity of this specification; it should not be read as an observed sudden increase at Day 7.

Of the 247 children, 42 were excluded from adjusted analyses because at least one model covariate was missing. Missing values occurred in dressing-change count (6 children), painful-procedure count (10), physiotherapy-session count (19) and local complications (11), with overlap. Covariate coefficients are conditional associations with average score level, not evidence that these factors altered the trajectory or caused symptom persistence.

The candidate comparison favored Day 2 over Day 7: AIC was −5342.5 versus −4929.6 (difference 412.8) for the discontinuous models. Day 3 had an AIC of −5331.7, and Day 8 had an AIC of −4812.0. The continuous model family also favored Day 2 (AIC −5326.5), compared with −4750.4 at Day 7. At the selected Day-2 breakpoint, the post-break slope in the discontinuous model was −0.0006/day (95% CI −0.0015 to 0.0003). These comparisons support an early change in the mean curve within this dataset, not an independently validated clinical reassessment time.

### 3.7. Sensitivity Analysis

The Gaussian GEE with exchangeable working correlation gave a post-Day-7 slope of +0.0002/day (95% CI −0.0004 to +0.0009; *p* = 0.455; Table 7). Agreement with the fixed-break mixed model does not establish that Day 7 is the best breakpoint. Unweighted GEE also does not resolve bias caused by outcome-dependent observation.

### 3.8. Exploratory Analysis of Therapy Modifications

In the exploratory switch model, the estimated level change after modification was +0.0208 normalized units (95% CI −0.0015 to +0.0430; *p* = 0.067), and the change in slope was +0.0001/day (−0.0032 to +0.0034; *p* = 0.955; Table 8). These estimates do not show a clear association in this dataset. Their uncertainty and confounding by the indication for switching preclude a conclusion that treatment modification has no effect.

### 3.9. Missing Data

There were 50 missing daily scores (20.2%) on Day 14. The missingness model included 3013 transitions with an observed preceding-day score; 424 were followed by a missing assessment. Mean preceding normalized itch was 0.608 before a missing assessment and 0.635 before an observed assessment. Missingness increased with follow-up day (odds ratio 1.10 per day, 95% CI 1.07–1.13). The preceding score was not clearly associated after adjustment (odds ratio 0.67 per full normalized unit, 0.32–1.37; *p* = 0.269). This does not establish MAR, particularly because transitions after missing days were excluded. Under the extreme minimum/maximum assignments, the Day-14 mean could range from 0.481 to 0.684, compared with the observed mean of 0.604. Later symptom burden remains sensitive to unobserved outcomes.

## 4. Discussion

The principal finding is an early fall in reconstructed itch scores followed by little change in the population mean, while symptoms remained present in every observed assessment. This pattern is consistent with little further change in the observed mean pruritus score during later follow-up, rather than documented symptom resolution. It does not establish continuous symptoms on unobserved days or persistence beyond Day 14. The original stored plateau field did not agree with events reconstructed using explicit daily-score rules. Their timing changed with the requirement for prior improvement or a longer unchanged sequence. A flat late slope in a fixed-Day-7 model does not identify the onset of either an individual operational score-stability event or clinical stabilization.

The study cannot attribute the early decrease to antihistamines. Treatment began several days after injury, while healing, inflammation and other aspects of care were changing. Regression to the mean may also contribute when treatment is initiated because symptoms are troublesome. Published work describes interacting neural and immune pathways in itch, including experimental evidence linking wound itch to cytokine signaling [3,12,13]. No measurements in this study identify a transition from histaminergic to neuropathic mechanisms. The observed time course should therefore remain a descriptive finding.

Repeated nursing assessment remains important for documenting itch, sleep disruption and interference with care. These data do not establish when to stop, prolong or change an antihistamine, nor when to introduce another agent. Evidence concerning alternative interventions addresses a separate treatment question [4,5]. A prospective study should use standardized instruments from a common clinical time point, record concurrent care and compare treatment strategies before testing a particular reassessment schedule.

Concurrent wound care is relevant to interpretation. Nicolosi et al. described variation in early pediatric burn-care practices across Italian centers, while Liu et al. highlighted the range of therapeutic approaches involved in the management of pediatric deep partial-thickness burn wounds [14,15]. Landino et al., van de Warenburg et al. and Rashaan et al. documented heterogeneity in pediatric burn-dressing strategies and related outcomes, including procedural pain and dressing burden [16,17,18,19]. In a separate retrospective cohort of mild-to-moderate burns, Nicolosi et al. reported associations between early enzyme–alginogel treatment and healing, complications and care burden [20]. These studies concern care practices and wound outcomes; they do not establish an antipruritic effect or a reassessment interval in the present cohort. They underscore the need to record concurrent interventions before attempting to separate their effects.

The strong age/tool interaction also argues against presenting normalized IMS and TPIS scores as interchangeable. Differences may reflect age, reporting method, scale properties or their combination. Separate original-scale analyses retain those distinctions, although retrospective reconstruction remains a limitation in both groups.

The alternative operational score-stability rules illustrate the effect of coarse ordinal categories. Repeated identical scores can occur without improvement, and a short unchanged sequence can be followed by worsening. Missing days further influence the first confirmable event. Day 4 is the conditional median among the 177 children meeting a post hoc rule; Day 2 is the best-fitting population breakpoint among the candidates examined. Neither identifies an optimal nursing reassessment interval. No alternative reassessment schedules or clinical consequences of earlier versus later review were compared. All reported days are measured from antihistamine initiation, not from burn injury. An operational score-stability event at a positive score must be distinguished from symptom resolution and should not be interpreted as sustained clinical stabilization.

### Strengths and Limitations

Strengths include repeated daily records and the ability to examine the same observations under explicit alternative definitions. The revised analyses separate individual score sequences from population-level curve fitting and report the actual sample contributing to adjusted models. Their value is exploratory; model agreement cannot compensate for uncertain measurement or the absence of a comparison group.

The single-center setting limits transportability. Referral patterns, local prescribing and dressing practices, staff expertise, and the attention given to itch may influence both care and the frequency or detail of documentation. Heightened awareness could increase detection and recording, but staff awareness was not measured, so its contribution and direction cannot be estimated. A multicenter study might produce different levels or trajectories because of differences in case mix, care and measurement; whether it would do so cannot be determined here. The findings should be interpreted as experience from this hospital and require external replication before application to other settings.

Scores were reconstructed from narrative records; independent pre-consensus ratings were not collected, and consensus review cannot establish coding reliability. Translation and review by five experts do not establish Italian psychometric or cross-cultural validity. The ordinal frameworks differ in age group and assessment approach, and linear models approximate their bounded outcomes. Residual confounding includes unmodelled anatomical sites and healing phase, grafts, xerosis, topical care and detailed analgesic exposure. Several included covariates summarize follow-up care and may themselves be consequences of the clinical course. Complete-covariate analysis excluded 42 children, while later outcome missingness could depend on unobserved symptoms. The missingness model cannot verify MAR, and absence of recorded zero scores does not exclude resolution on unobserved days. Revised operational score-stability rules and breakpoint selection were exploratory; conditional confidence intervals do not capture all model-selection uncertainty.

## 5. Conclusions

In this single-center cohort, reconstructed itch scores decreased early following antihistamine initiation and subsequently showed little change in the mean, with residual pruritus in all observed assessments through Day 14. Day 4 was the median confirmation day only among the 177 children meeting a post hoc stability rule. The data describe temporal evolution under combined routine care; they do not separate antihistamine effects from nursing interventions or identify an optimal reassessment interval. Prospective multicenter studies should use appropriately adapted and validated measures, record concurrent care, and compare reassessment strategies against clinically meaningful outcomes.

## Figures and Tables

**Table 1:** Baseline characteristics of the study population (N = 247).

Characteristic	Overall (N = 247)
Age, years	9.62 ± 4.86
Male sex, n (%)	130 (52.6)
Weight, kg	38.07 ± 17.48
TBSA, %	27.05 ± 10.92
Burn Depth, n (%)	
Superficial partial thickness	141 (57.1)
Deep partial thickness	75 (30.4)
Full thickness	31 (12.6)
Burn Etiology, n (%)	
Thermal	200 (81.0)
Chemical	37 (15.0)
Electrical	10 (4.0)
Burn Site, n (%)	
Head and neck	101 (40.9)
Trunk	102 (41.3)
Upper limbs	92 (37.2)
Lower limbs	95 (38.5)
Hands and feet	103 (41.7)
Genital and perineal area	116 (47.0)
Days from burn to antihistamine initiation	5.37 ± 2.47

Note: Patients could present burns involving multiple anatomical sites; therefore, percentages for burn location do not sum to 100%. TBSA = total body surface area.

**Table 2:** Recorded initial antihistamine therapy, dose and frequency.

Initial Drug	n (%)	Frequency	Daily Dose, mg Mean ± SD	mg/kg/Day Mean ± SD
Levocetirizine	63 (25.5)	QD	3.12 ± 1.57	0.084 ± 0.024
Desloratadine	50 (20.2)	QD	2.73 ± 1.59	0.083 ± 0.026
Cetirizine	45 (18.2)	QD	7.61 ± 3.20	0.221 ± 0.079
Loratadine	35 (14.2)	QD	8.71 ± 2.22	0.258 ± 0.080
Hydroxyzine	29 (11.7)	TID	60.63 ± 15.42	1.544 ± 0.395
Chlorpheniramine	25 (10.1)	QID	12.72 ± 6.45	0.287 ± 0.066

Note: Values describe the database and do not constitute dosing recommendations. QD = once daily; TID = three times daily; QID = four times daily.

**Table 3:** Treatment exposure and therapy modification.

Exposure Summary	Value
Treatment duration, days	11.45 ± 1.69
Cumulative doses, count	18.29 ± 13.89
Switch or add-on therapy, n (%)	45 (18.2)

Note: Switch/add-on indicates any change in antihistamine regimen during follow-up.

**Table 4:** Daily itch trajectory (normalized) and missingness across follow-up.

Day	Observed, n	Missing, n (%)	Normalized Itch (Mean ± SD)
0	247	0 (0.0)	0.836 ± 0.139
1	235	12 (4.9)	0.700 ± 0.153
2	223	24 (9.7)	0.628 ± 0.141
3	214	33 (13.4)	0.605 ± 0.116
4	227	20 (8.1)	0.601 ± 0.115
5	222	25 (10.1)	0.595 ± 0.113
6	218	29 (11.7)	0.591 ± 0.113
7	216	31 (12.6)	0.603 ± 0.131
8	210	37 (15.0)	0.608 ± 0.140
9	212	35 (14.2)	0.603 ± 0.139
10	197	50 (20.2)	0.607 ± 0.141
11	192	55 (22.3)	0.606 ± 0.139
12	199	48 (19.4)	0.603 ± 0.138
13	201	46 (18.6)	0.604 ± 0.136
14	197	50 (20.2)	0.604 ± 0.136

Note: Normalized itch score ranges from 0 to 1 (IMS/4; TPIS/3). IMS = Itch Man Scale; TPIS = Toronto Pediatric Itch Scale.

**Table 5:** Post hoc sensitivity analysis of individual operational score-stability events.

Operational Score-Stability Rule	Observed Events; Confirmation Day
Three unchanged daily scores	247/247 (100%); median 3 (IQR 2–4.5)
Three unchanged scores after improvement	177/247 (71.7%); median 4 (IQR 3–5)
Four unchanged scores after improvement	171/247 (69.2%); median 5 (IQR 4–7)
Three unchanged positive scores after improvement	177/247 (71.7%); median 4 (IQR 3–5)

Note: Event day is the confirmation day. Medians and IQRs refer only to children meeting each rule; percentages use all 247 children.

**Table 6:** Recalculated mixed-effects model with a fixed Day-7 breakpoint (2654 observations; 205 children).

Predictor	Beta (SE)	95% CI	*p*-Value
Intercept	0.7904 (0.0384)	0.7151 to 0.8656	<0.001
Slope before Day 7	−0.0358 (0.0012)	−0.0381 to −0.0335	<0.001
Level change at Day 7	0.0932 (0.0068)	0.0799 to 0.1066	<0.001
Change in slope after Day 7	0.0361 (0.0016)	0.0330 to 0.0391	<0.001
Burn depth: superficial	0.0075 (0.0150)	−0.0219 to 0.0369	0.619
Burn depth: full thickness	0.0234 (0.0230)	−0.0217 to 0.0685	0.310
Etiology: electrical	0.0073 (0.0357)	−0.0626 to 0.0773	0.837
Etiology: thermal	0.0417 (0.0192)	0.0042 to 0.0793	0.029
Sex: male	−0.0143 (0.0129)	−0.0397 to 0.0111	0.269
Age (years)	−0.0257 (0.0074)	−0.0403 to −0.0111	<0.001
Dressing-change count	0.0033 (0.0025)	−0.0016 to 0.0081	0.190
Local complications	−0.0117 (0.0201)	−0.0511 to 0.0277	0.562
Opioid use	0.0202 (0.0152)	−0.0097 to 0.0501	0.185
Painful-procedure count	−0.0015 (0.0050)	−0.0113 to 0.0083	0.758
Physiotherapy-session count	−0.0025 (0.0019)	−0.0062 to 0.0011	0.174
Sedation/drowsiness	0.0051 (0.0171)	−0.0285 to 0.0387	0.766
TBSA (%)	−0.0012 (0.0009)	−0.0030 to 0.0006	0.196
Weight (kg)	0.0048 (0.0020)	0.0008 to 0.0088	0.018
Post-Day-7 slope (sum)	0.0002 (0.0010)	−0.0018 to 0.0022	0.817

Note: Coefficients refer to normalized scores. The final row is a derived linear combination, not an additional fitted term.

**Table 7:** Gaussian GEE at a fixed Day-7 breakpoint, with patient-level robust standard errors (2654 observations; 205 children).

Predictor	Beta (SE)	95% CI	*p*-Value
Intercept	0.7904 (0.0353)	0.7212 to 0.8595	<0.001
Slope before Day 7	−0.0358 (0.0021)	−0.0399 to −0.0317	<0.001
Level change at Day 7	0.0932 (0.0058)	0.0819 to 0.1046	<0.001
Change in slope after Day 7	0.0361 (0.0021)	0.0319 to 0.0402	<0.001
Burn depth: superficial	0.0075 (0.0149)	−0.0217 to 0.0366	0.616
Burn depth: full thickness	0.0234 (0.0211)	−0.0180 to 0.0648	0.268
Etiology: electrical	0.0073 (0.0313)	−0.0539 to 0.0686	0.815
Etiology: thermal	0.0417 (0.0156)	0.0112 to 0.0723	0.007
Sex: male	−0.0143 (0.0129)	−0.0395 to 0.0109	0.266
Age (years)	−0.0257 (0.0070)	−0.0393 to −0.0120	<0.001
Dressing-change count	0.0033 (0.0024)	−0.0014 to 0.0079	0.169
Local complications	−0.0117 (0.0188)	−0.0486 to 0.0253	0.536
Opioid use	0.0202 (0.0161)	−0.0114 to 0.0518	0.210
Painful-procedure count	−0.0015 (0.0049)	−0.0111 to 0.0080	0.753
Physiotherapy-session count	−0.0025 (0.0018)	−0.0061 to 0.0011	0.167
Sedation/drowsiness	0.0051 (0.0186)	−0.0314 to 0.0416	0.784
TBSA (%)	−0.0012 (0.0009)	−0.0030 to 0.0006	0.192
Weight (kg)	0.0048 (0.0020)	0.0010 to 0.0086	0.014
Post-Day-7 slope (sum)	0.0002 (0.0003)	−0.0004 to 0.0009	0.455

Note: The final row reports the derived post-break slope.

**Table 8:** Exploratory mixed-effects model adding switch-related level and slope changes to the fixed-Day-7 specification.

Predictor	Beta (SE)	95% CI	*p*-Value
Slope before Day 7	−0.0365 (0.0012)	−0.0389 to −0.0341	<0.001
Level change at Day 7	0.0935 (0.0068)	0.0801 to 0.1068	<0.001
Change in slope after Day 7	0.0367 (0.0016)	0.0335 to 0.0399	<0.001
Level change after switch	0.0208 (0.0114)	−0.0015 to 0.0430	0.067
Change in slope after switch	0.0001 (0.0017)	−0.0032 to 0.0034	0.955

Note: Other covariates were retained. Switching was not randomized.

## Data Availability

The data presented in this study are available from the corresponding author upon reasonable request. The data are not publicly available because they contain potentially identifiable clinical information.

## Supplementary Materials

The following supporting information can be downloaded at https://www.mdpi.com/article/10.3390/medsci14060634/s1: Supplementary File S1, Retrospective pruritus coding framework; Supplementary File S2, Illustrative coding examples and quality control; and Supplementary File S3, Statistical appendix for the revised analyses.

## Abbreviations

The following abbreviations are used in this manuscript:

AIC	Akaike information criterion
CI	Confidence interval
GDPR	General Data Protection Regulation
GEE	Generalized estimating equations
IMS	Itch Man Scale
IQR	Interquartile range
MAR	Missing at random
MCID	Minimal clinically important difference
QD	Once daily
QID	Four times daily
REDCap	Research Electronic Data Capture
SD	Standard deviation
SE	Standard error
STROBE	Strengthening the Reporting of Observational Studies in Epidemiology
TBSA	Total body surface area
TID	Three times daily
TPIS	Toronto Pediatric Itch Scale
