# Supplementary material for: Persistent Post-Burn Pruritus Following Antihistamine Initiation: A Retrospective Longitudinal Study at a Single Italian Pediatric Burn Center
Source: Med Sci (Basel). 2026 Oct 2;14(6):634. doi: 10.3390/medsci14060634 (PMC13706701; doi:10.3390/medsci14060634)
Supplement: Supplementary file 1 [file medsci-14-00634-s001.zip › Supplementary_File_S1.pdf]

Supplementary File S1. Retrospective pruritus coding framework

This supplement describes the study-specific algorithm used to reconstruct pruritus categories from routine medical and nursing records. The algorithm was developed before data extraction. The category descriptors below reproduce that coding framework; they are not the wording of prospectively administered IMS or TPIS instruments. The clinical data came from patient charts. This retrospective mapping has not been independently validated.

The source IMS and TPIS instruments have validation evidence from English-language settings. For local use, they were translated into Italian, with linguistic adaptation and a final review by five experts. This process addressed the language version; it did not validate the retrospective assignment of scores or establish the Italian versions' reliability, responsiveness or cross-cultural measurement equivalence. The descriptors below remain the study-specific retrospective framework. References to the original scale studies and the methodological distinction between adaptation and validation are provided in Section 2.3 of the manuscript.

General principles

- Children ≥ 6 years were classified according to the Itch Man Scale (IMS); children < 6 years according to the Toronto Pediatric Itch Scale (TPIS).
- Only narrative notes with internally consistent information were eligible for coding.
- If several descriptors belonging to different severity categories were present, the highest category was assigned only when supported by the overall clinical narrative.
- Incomplete or contradictory documentation was coded as missing.
- When multiple assessments were available on the same day, the highest score was retained (worst-of-day criterion).

Table S1.1. IMS coding framework

Score	Severity	Descriptors
0	None	No itch reported; no scratching; comfortable behaviour.
1	Mild	Occasional itch, isolated scratching, no sleep or activity impairment.
2	Moderate	Frequent scratching, discomfort, occasional sleep disturbance, reassurance required.
3	Severe	Persistent itch affecting sleep or daily activities; repeated nursing intervention.
4	Very severe	Continuous scratching, major sleep disruption, dressing disruption, repeated requests for relief.

Table S1.2. TPIS coding framework

Score	Severity	Descriptors
0	None	No behavioural evidence of itching.
1	Mild	Occasional rubbing; easily consolable.
2	Moderate	Frequent rubbing/scratching, irritability, disturbed sleep.
3	Severe	Persistent scratching, inconsolable crying, repeated nursing intervention.

Decision workflow

Clinical record → Determine age group → Verify sufficient documentation → Identify behavioural/clinical descriptors → Assign IMS or TPIS category → If multiple observations on same day retain worst score → Normalize (IMS/4 or TPIS/3) for secondary pooled summaries only.

Measurement limitations

The framework standardizes the interpretation of narrative documentation but cannot recover information that was not recorded. The zero category describes documented absence of itch or relevant behaviour; lack of usable documentation is classified as missing under the rules above. In the analysed data, no observed score was zero. Normalization is used only for secondary pooled analyses and does not demonstrate equivalence between the two frameworks. Independent pre-consensus ratings were not collected, so coding reproducibility cannot be quantified. Supplementary File S2 clarifies the review procedure; Supplementary File S3 describes analyses introduced during revision.
