# Supplementary material for: Persistent Post-Burn Pruritus Following Antihistamine Initiation: A Retrospective Longitudinal Study at a Single Italian Pediatric Burn Center
Source: Med Sci (Basel). 2026 Oct 2;14(6):634. doi: 10.3390/medsci14060634 (PMC13706701; doi:10.3390/medsci14060634)
Supplement: Supplementary file 1 [file medsci-14-00634-s001.zip › Supplementary_File_S2.pdf]

**Supplementary File S2. Illustrative coding examples and quality control**

The six examples below are retained from the original illustrative coding manual. They demonstrate the mapping in Supplementary File S1 and are not presented as an independent validation sample or as verified verbatim extracts from individual patient charts. Their category assignments have not been changed.

**Example 1**

Clinical note:

Occasional itching reported during dressing change. Child slept well overnight.

Assigned score: IMS 1

**Example 2**

Clinical note:

Frequent scratching requires reassurance; mild sleep disturbance.

Assigned score: IMS 2

**Example 3**

Clinical note:

Persistent scratching with repeated nursing intervention and impaired sleep.

Assigned score: IMS 3

**Example 4**

Clinical note:

Continuous scratching resulting in dressing displacement.

Assigned score: IMS 4

**Example 5**

Clinical note:

Infant occasionally rubbing wounds but easily comforted.

Assigned score: TPIS 1

**Example 6**

Clinical note:

Infant persistently rubbing wound, inconsolable, repeated intervention.

Assigned score: TPIS 3

**Quality assurance**

Retrospective score attribution was performed by an experienced wound-care nurse. A second investigator reviewed coding ambiguities, which were resolved by consensus. This review served as a quality-control step to support consistent application of the coding framework; it was not a formal independent reliability study. Separate pre-consensus ratings were not collected. Consequently, weighted kappa and its confidence interval cannot be calculated, and the reproducibility of score reconstruction remains unquantified. No new independent recoding was possible during revision. The five-expert review of the Italian language versions was a separate adaptation step; it did not provide independent patient-level ratings or a measure of inter-rater agreement.
