# Supplementary material for: Persistent Post-Burn Pruritus Following Antihistamine Initiation: A Retrospective Longitudinal Study at a Single Italian Pediatric Burn Center
Source: Med Sci (Basel). 2026 Oct 2;14(6):634. doi: 10.3390/medsci14060634 (PMC13706701; doi:10.3390/medsci14060634)
Supplement: Supplementary file 1 [file medsci-14-00634-s001.zip › Supplementary_File_S3.pdf]

**Supplementary File S3. Statistical appendix for the revised analyses**

This appendix documents the analyses performed during manuscript revision. The revised stability definitions, breakpoint comparisons, age/tool interaction, separate framework-specific models and missingness analyses are exploratory and were not prespecified. They describe an early reduction followed by persistent recorded itch; they do not estimate a causal treatment effect or validate a clinical reassessment time.

**S3.1 Data and analysis populations**

All 247 available records for the study population were included without sampling a subset. The clinical dataset contains 247 children and 3,705 potential child-day records from Day 0 to Day 14 following antihistamine initiation. There are 3,210 observed itch scores and 495 missing scores (13.4%). All observed scores are positive, with a minimum recorded category of 2. IMS-derived categories apply to 181 children and TPIS-derived categories to 66. Stored segmentation variables and the stored plateau estimate were not used to define the revised events or candidate breakpoints.

Descriptive analyses use all available outcomes. Adjusted longitudinal models use 2,654 observed scores from 205 children with complete model covariates. Forty-two children are excluded because at least one covariate is missing. Missing covariate counts are 6 for dressing changes, 10 for painful procedures, 19 for physiotherapy sessions and 11 for local complications; these groups overlap. Outcomes and covariates were not imputed for regression.

**S3.2 Daily summaries and persistence**

For each day, means and standard deviations use available scores. Pointwise 95% confidence intervals for plotted means are mean ± 1.96 × SD/√n. Native IMS-derived and TPIS-derived summaries are reported separately. Secondary normalized values equal IMS/4 or TPIS/3. Equal normalized values do not imply equal clinical severity.

**Table S3.1** Daily observation counts and native-scale means

| Day | Observed n | Missing n (%) | IMS mean (n) | TPIS mean (n) |
|---|---|---|---|---|
| 0 | 247 | 0 (0.0) | 3.44 (181) | 2.32 (66) |
| 1 | 235 | 12 (4.9) | 2.75 (169) | 2.20 (66) |
| 2 | 223 | 24 (9.7) | 2.40 (165) | 2.12 (58) |
| 3 | 214 | 33 (13.4) | 2.30 (154) | 2.05 (60) |
| 4 | 227 | 20 (8.1) | 2.29 (167) | 2.05 (60) |
| 5 | 222 | 25 (10.1) | 2.24 (160) | 2.05 (62) |
| 6 | 218 | 29 (11.7) | 2.23 (162) | 2.05 (56) |
| 7 | 216 | 31 (12.6) | 2.24 (157) | 2.15 (59) |
| 8 | 210 | 37 (15.0) | 2.24 (154) | 2.21 (56) |
| 9 | 212 | 35 (14.2) | 2.21 (154) | 2.21 (58) |
| 10 | 197 | 50 (20.2) | 2.22 (141) | 2.21 (56) |
| 11 | 192 | 55 (22.3) | 2.22 (138) | 2.20 (54) |

| Day | Observed n | Missing n (%) | IMS mean (n) | TPIS mean (n) |
|---|---|---|---|---|
| 12 | 199 | 48 (19.4) | 2.21 (145) | 2.20 (54) |
| 13 | 201 | 46 (18.6) | 2.23 (147) | 2.19 (54) |
| 14 | 197 | 50 (20.2) | 2.23 (144) | 2.19 (53) |

All observed assessments in every row of Table S3.1 have scores above zero. In particular, 197/197 available Day-14 assessments are positive, representing 197/247 children (79.8% of the cohort); the status of the remaining 50 children on that day is unknown. A positive observed score documents residual symptoms at that assessment, not uninterrupted symptoms between assessments or persistence beyond follow-up.

**S3.3 Individual stability events**

Let s(t) be the native ordinal score on calendar day t after initiation. Rule A is first confirmed on day $t \geq 2$ if $s(t-2)$, $s(t-1)$ and $s(t)$ are all observed and identical. Rule B adds $s(0)-s(t) \geq 1$. Rule C requires four observed identical scores on days $t-3$ through t, together with $s(0)-s(t) \geq 1$. The positive-score variant of B additionally requires $s(t)>0$. The event day is the last day needed to confirm the sequence. Improvement is relative to Day 0 and need not be monotonic beforehand.

An unchanged sequence without prior improvement qualifies only for A. Missing observations interrupt the sequence; nonconsecutive days are not joined. A worsening transition breaks an unchanged sequence, but a later sequence may qualify. Low unchanged scores qualify for A, while B and C still require at least one category of baseline improvement. Later worsening does not revoke an event. A child without a qualifying observed sequence by Day 14 is classified as having no observed event, rather than assumed never to stabilize. None of these rules is an MCID or a validated clinical endpoint.

**Table S3.2** Sensitivity to the post hoc stability definition

| Rule | Events n/N (%) | No event n | Day median (IQR) |
|---|---|---|---|
| A Three unchanged scores | 247/247 (100.0) | 0 | 3 (2–4.5) |
| B Three after improvement | 177/247 (71.7) | 70 | 4 (3–5) |
| C Four after improvement | 171/247 (69.2) | 76 | 5 (4–7) |
| B with positive scores | 177/247 (71.7) | 70 | 4 (3–5) |

Medians and IQRs are calculated only among children meeting the relevant rule, using linear interpolation for quantiles. Day 4 is therefore the conditional median among 177 children meeting B, not a cohort-wide threshold. The stored plateau day agrees with A in 4/247 children and B in 6/247, so it cannot be treated as the same endpoint. Figure 2 uses all 247 children as its denominator at each day; it is an empirical cumulative event proportion, not a Kaplan–Meier estimate.

**S3.4 Segmented mixed-effects models**

The secondary pooled outcome y is the normalized score. A Gaussian linear mixed-effects model with a patient-specific random intercept was fitted by maximum likelihood. For candidate breakpoint k, the fixed time terms are day t, post = $I(t \geq k)$, and hinge = $\max(0,t-k)$.

$$y(i,t) = \beta0 + \beta1\, t + \beta2\, I(t \geq k) + \beta3 \max(0,t-k) + \gamma X(i) + b(i) + \varepsilon(i,t)$$

X contains age in years, sex, weight, TBSA percentage, burn depth, burn etiology, opioid use, sedation/drowsiness, dressing-change count, painful-procedure count, physiotherapy-session count and local infection/complications. Reference categories are female sex, deep partial-thickness burns and chemical etiology. These adjustment variables are patient-level characteristics or follow-up care summaries, not daily time-varying exposures. Adjustment for follow-up summaries may introduce bias and does not establish causal effects.

Covariate coverage was incomplete. TBSA and depth were included. Anatomical site indicators and time to re-epithelialization were available but were not included in these models; daily healing phase was not represented. Dressing-change, painful-procedure and physiotherapy counts were aggregate care measures. The infection/complication variable was a combined indicator rather than a dated, microbiologically defined infection measure. Analgesic control comprised opioid use and sedation/drowsiness, without daily dose or timing. Grafting, donor-site itch, xerosis, topical products, skin hydration, comfort measures and education were not available as structured analysis exposures. These factors remain possible sources of residual confounding; routine clinical standards do not establish that they were measured or controlled in this analysis.

$\beta 1$ is the pre-break slope; $\beta 2$ is a permitted level discontinuity; $\beta 3$ is the change in slope. The post-break slope is $\beta 1+\beta 3$. Its standard error is $\sqrt{[\mathrm{Var}(\beta 1)+\mathrm{Var}(\beta 3)+2\mathrm{Cov}(\beta 1,\beta 3)]}$, using the fitted covariance matrix. The 95% CI is the sum ±1.96 times its standard error. A non-significant late slope does not prove exact stability. The discontinuity does not represent an observed abrupt biological event.

The original fixed-Day-7 specification is retained for comparison. Tables 6 and 7 in the manuscript report the complete fixed effect estimates for the mixed model and Gaussian GEE. The random-intercept variance for the Day-7 mixed model is 0.007507. Maximum-likelihood fitting used the Powell optimizer; an independent BFGS refit of the Day-7 model converged to essentially identical coefficients.

**S3.5 Candidate breakpoints and age or tool differences**

Discontinuous models were compared at Days 2–12 using identical covariates and observations. Day 1 is not identifiable under this specification because there is only one pre-break time point. Continuous models omit the level-change term and were compared at Days 1–12. AIC values are conditional model-fit comparisons; they are not confidence intervals for breakpoint location. Selection uncertainty is not included in a selected model's coefficient intervals.

**Table S3.3** Candidate breakpoint model fit

| Breakpoint day | Discontinuous AIC | Continuous AIC |
|---|---|---|
| 1 | Not identifiable | -5175.8 |
| 2 | -5342.5 | -5326.5 |
| 3 | -5331.7 | -5245.7 |
| 4 | -5255.8 | -5104.8 |
| 5 | -5133.3 | -4976.0 |
| 6 | -5023.4 | -4859.2 |
| 7 | -4929.6 | -4750.4 |
| 8 | -4812.0 | -4663.1 |
| 9 | -4712.9 | -4592.5 |
| 10 | -4628.1 | -4535.7 |
| 11 | -4558.8 | -4489.2 |
| 12 | -4500.9 | -4449.9 |

Both model families favor Day 2. In the discontinuous family, the AIC difference between Day 7 and Day 2 is 412.8. The selected Day-2 post-break slope is −0.0006/day (95% CI −0.0015 to 0.0003). This describes an early change in the population mean, distinct from the confirmation day of an individual stability event.

A Gaussian GEE with exchangeable working correlation and patient-level robust standard errors included the three interactions of tool with day, post and hinge at Day 7. Their joint Wald test gave $\chi^2$=54.54 with 3 degrees of freedom ($p<0.001$). Because age determines tool assignment, this is an age/tool contrast and cannot isolate an instrument effect. Separate mixed models used original-scale scores and the same covariates.

**Table S3.4** Native-scale temporal coefficients at the fixed Day-7 breakpoint

| Framework | Term | Estimate | 95% CI | p |
|---|---|---|---|---|
| IMS | Pre-break slope | -0.1731 | -0.1829 to -0.1633 | <0.001 |
| IMS | Level change | 0.3918 | 0.3351 to 0.4484 | <0.001 |
| IMS | Change in slope | 0.1734 | 0.1604 to 0.1863 | <0.001 |
| IMS | Post-break slope | 0.0003 | -0.0082 to 0.0088 | 0.951 |
| TPIS | Pre-break slope | -0.0429 | -0.0565 to -0.0293 | <0.001 |
| TPIS | Level change | 0.2379 | 0.1591 to 0.3168 | <0.001 |
| TPIS | Change in slope | 0.0444 | 0.0264 to 0.0624 | <0.001 |
| TPIS | Post-break slope | 0.0015 | -0.0101 to 0.0132 | 0.796 |

IMS: 153 children and 1,968 observations. TPIS: 52 children and 686 observations. Coefficients are categories/day for slope terms and categories for level terms. Pooled rescaling remains a secondary summary despite these additional analyses.

**S3.6 GEE and treatment modification**

The pooled Gaussian GEE uses the fixed-Day-7-time terms and the same complete-covariate population. Its post-break slope is 0.0002/day (95% CI −0.0004 to 0.0009; p=0.455). Robust standard errors address within-child dependence but do not resolve outcome-dependent observation. A separate mixed model adds an indicator for observations on or after the recorded switch/add-on day and elapsed time after that day; both terms are zero for children without modification. Table 8 reports these estimates. Switching was not randomized, and the analysis cannot establish that modifying treatment is ineffective.

**S3.7 Missingness analyses**

For Days 1–14, a logistic GEE with independence working correlation and patient-level robust standard errors models whether the current score is missing. It includes only transitions with an observed immediately preceding calendar day. Predictors are day, preceding normalized score, age, sex, TBSA and burn depth. The analysis contains 3,013 transitions, including 424 missing current scores and 2,589 observed current scores. Mean preceding itch is 0.608 before missing and 0.635 before observed assessments. Exclusion of transitions after missing days limits this analysis.

Table S3.5 Adjusted associations with a missing current score

| Predictor | Odds ratio | 95% CI | p |
|---|---|---|---|
| Day (per day) | 1.102 | 1.073 to 1.132 | <0.001 |
| Previous score (per 0–1 unit) | 0.667 | 0.325 to 1.368 | 0.269 |
| Age (per year) | 1.013 | 0.991 to 1.036 | 0.248 |
| TBSA (per percentage point) | 0.996 | 0.984 to 1.008 | 0.472 |
| Male vs female | 0.996 | 0.812 to 1.221 | 0.969 |
| Superficial vs deep partial thickness | 1.364 | 1.079 to 1.724 | 0.009 |
| Full vs deep partial thickness | 0.995 | 0.636 to 1.556 | 0.982 |

Missingness increases with day. The preceding observed score is not clearly associated after adjustment, but this does not demonstrate that missing outcomes are missing at random. No observed-data test can exclude dependence on the unobserved current score.

Extreme descriptive bounds assign each missing normalized score first 0 and then 1, retaining all 247 children in the denominator. The lower mean is the sum of observed normalized scores divided by 247; the upper mean adds the number missing to that numerator. At Day 14 these means are 0.481 and 0.684, compared with the available-case mean of 0.604. These are bounds under extreme assignments, not confidence intervals or model-based imputations. They show sensitivity to unobserved outcomes and do not establish robustness to all missingness mechanisms.

**S3.8 Interpretation and software**

The analyses used Python 3.12, pandas, SciPy and statsmodels 0.15.0. Tests are two-sided with 95% confidence intervals; no multiplicity adjustment was made. Linear models approximate bounded ordinal scores. All added analyses are exploratory. The absence of an untreated comparison group, retrospective measurement without quantified inter-rater agreement, incomplete outcomes and complete-covariate selection remain limitations. Neither Day 2 nor Day 4 is proposed as a validated clinical threshold. These data do not compare reassessment schedules and cannot identify an

optimal nursing review interval or separate pharmacological and nursing contributions to symptom change.
